# Commentary: Pemphigoid associated with quinolone antibiotics: a real-world pharmacovigilance study of the FDA adverse event reporting system and literature-based evidence

**DOI:** 10.3389/fimmu.2026.1873163

**Published:** 2026-07-17

**Authors:** Hai-Hong Lin, Kai-Li Mao, Liu-Cheng Li, Huan-Hao Zhou

**Affiliations:** 1 Department of Pharmacy, Wenling TCM Hospital Affiliated to Zhejiang Chinese Medical University, Wenling, China; 2 Department of Pharmacy, The Quzhou Affiliated Hospital of Wenzhou Medical University, Quzhou People’s Hospital, Quzhou, China; 3 Department of Pharmacy, Sir Run Run Shaw Hospital, Zhejiang University School of Medicine, Hangzhou, China; 4 Department of Thyroid and Breast Surgery, The Quzhou Affiliated Hospital of Wenzhou Medical University, Quzhou People’s Hospital, Quzhou, China

**Keywords:** FAERS, immune-related adverse drug reactions, pemphigoid, pharmacists, pharmacovigilance

## 1 Introduction

Li and colleagues (2026) have recently conducted the first systematic pharmacovigilance analysis linking quinolone antibiotics to pemphigoid using the FDA Adverse Event Reporting System (FAERS) and published literature (1). The authors report significant disproportionality signals for ofloxacin, levofloxacin, and ciprofloxacin, with most events occurring within 30 days and a predominance in elderly females. These findings fill an important gap, as previous evidence had been limited to case reports, and they offer a clinically valuable alert for early monitoring during quinolone therapy.

However, the leap from a statistical reporting association to a clinically actionable causal inference requires several methodological bridges that the current study does not fully cross. The original study relies on a single spontaneous reporting database (FAERS) and only nine published case reports, inadequately presents key demographic and case-level details necessary for signal validation. The original study’s primary metric is the reporting odds ratio (ROR), a disproportionality measure that compares the odds of reporting pemphigoid for a specific quinolone versus all other drugs in the FAERS database. This commentary does not aim to dismiss the valuable signal but to critically reframe its interpretation, highlight unaddressed confounders, and introduce the systematically missing role of the clinical pharmacist, both in the study itself and in real-world pharmacovigilance practice.

This commentary does not present original pharmacovigilance data or conduct independent disproportionality analyses. Instead, our objectives are threefold to critically reframe the interpretation of the signal reported by Li et al. by highlighting unaddressed methodological confounders; to identify gaps in external validity and generalizability that limit translation of the signal into clinical practice; and to propose a clinically integrated roadmap that explicitly incorporates the role of clinical pharmacists, who are systematically missing from both the original study and current pharmacovigilance practice. The methodology of this commentary follows the established framework for critical academic commentaries in pharmacovigilance and clinical pharmacology. This approach is consistent with the standard structure of peer-reviewed commentaries in journals.

## 2 Unaddressed confounding

The authors acknowledge that they did not exclude cases with concomitant high-risk drugs such as DPP-4 inhibitors, aldosterone antagonists, anticholinergics, or immune checkpoint inhibitors, all well-established inducers of drug-induced pemphigoid (2–4). In an elderly population where polypharmacy is the rule rather than the exception, the observed ROR could be substantially inflated if a portion of cases were actually due to a co-administered drug. Without a sensitivity analysis excluding such cases, the specificity of the quinolone signal remains uncertain. Another notable limitation is the inability to distinguish between drug-induced and infection-induced pemphigoid. Infections themselves, particularly in the elderly, immunologically vulnerable patients, can trigger autoimmune blistering responses. A patient receiving a quinolone for a urinary or respiratory tract infection may develop pemphigoid not because of the drug, but due to the underlying infection activating an autoimmune diathesis. The authors touch on this but do not operationalize any analytic strategy (e.g., negative control antibiotics, self-controlled case series) to parse this critical confounding.

## 3 Overinterpretation of the literature-based evidence

The original study includes nine published case reports from 2000 to 2022 and presents them as an interaction analysis to provide an auxiliary clinical reference. The authors do not explicitly claim that these nine cases validate the FAERS signal. However, the small number of cases, inevitable given the rarity of the event, means that this literature evidence can only serve as supportive qualitative evidence, not as independent statistical validation. A more accurate description of the original study’s literature integration would be a qualitative consistency check rather than a confirmatory analysis. With such a tiny sample, this does not constitute external validation of signal strength or causality. It merely shows that the demographic and latency patterns in FAERS are not grossly inconsistent with the few well-documented cases. Nine case reports can serve as a qualitative consistency check, but they cannot validate a disproportionality signal. That requires independent cohort studies, nested case-control studies, or at minimum, replication in a second spontaneous reporting database. The authors appropriately note the small numbers. However, it should be aware that the term “interaction analysis” does not imply statistical validation; rather, the nine cases provide a qualitative consistency check that the FAERS patterns are not contradicted by published reports. We suggest that future studies explicitly label such literature comparisons as “supportive qualitative evidence” to avoid potential overinterpretation. To genuinely validate a FAERS signal, independent cohort studies or nested case-control studies are required.

## 4 External validity and generalizability

A fundamental methodological concern is that the original study does not explicitly adhere to established guidelines for pharmacovigilance disproportionality analysis, such as the READUS-PV (Reporting of A Disproportionality Analysis for Pharmacovigilance) statement. Key elements often required, including the handling of duplicate reports, specification of the background comparator, adjustment for multiple testing, and sensitivity analyses for concomitant medications, are incompletely reported. Future studies in this area should prospectively follow such guidelines to ensure transparency and reproducibility. FAERS is subject to underreporting, differential reporting by severity, and reporter type bias. While the authors stratify by reporter occupation, they do not quantify the potential impact of missing reports, nor do they compare reporting patterns between physicians vs pharmacists vs consumers yet. Notably, the original paper reports PH20 (10.9%) as pharmacist reports, a non-trivial proportion, yet the role of pharmacists is never discussed in the text (1). This represents a missed opportunity, because pharmacists are often the professional to detect potential adverse drug reactions (ADRs) during medication reconciliation or discharge counseling. No analysis of cumulative dose, treatment duration, or repeat exposure is provided. An immune-mediated reaction may not follow a simple dose-response relationship, but the complete absence of this information prevents any assessment of biological gradient, one of Hill’s criteria for causation (5).

Specifically, the original study does not report the demographics of individual case safety reports (ICSRs) in a manner that allows independent re-evaluation. Best practices for pharmacovigilance studies using FAERS recommend that ICSR-level data, including age (as continuous or categorical), sex, concomitant medications, drug indication, time-to-onset, de-challenge outcome, re-challenge outcome, and seriousness criteria. The original study provides only aggregate summaries, which obscures critical patterns such as whether hospitalized cases were predominantly elderly patients on DPP-4 inhibitors.

Furthermore, the original study inadequately presents granular demographic and case-level details that are essential for signal evaluation. While age stratification is provided, specific comorbid conditions, concomitant medication lists at the individual case level, and temporal relationships between drug initiation, symptom onset, and diagnosis are not systematically tabulated. Without such detailed case information, which is recommended in pharmacovigilance best practices, readers cannot independently assess potential confounding by polypharmacy or underlying disease. Besides, the original study provides no sensitivity analysis to validate the robustness of its reported 67.2% hospitalization rate (1). For example, does the hospitalization rate remain elevated after excluding cases with concomitant high-risk drugs known to cause pemphigoid (e.g., DPP-4 inhibitors, aldosterone antagonists)? Does it differ between patients with documented infection versus those without? Are there differences across quinolone types (ofloxacin vs. ciprofloxacin vs. levofloxacin) after adjusting for baseline comorbidities? Without such sensitivity analyses, the reported hospitalization rate is difficult to interpret as a drug-attributable risk. It may simply reflect the underlying illness burden (e.g., severe infection requiring admission) rather than a direct consequence of quinolone-induced pemphigoid.

The original study does not provide a pre-specified, hypothesis-driven rationale for which quinolones were analyzed. The stated rationale appears to be data-driven rather than hypothesis-driven, which increases the risk of type I error due to multiple testing. A more rigorous approach would have required pre-specification before analysis. More fundamentally, the original study does not adequately present the information of the quinolone-associated pemphigoid reaction within the FAERS database. Key case-level details that are essential for signal interpretation such as individual-level concomitant medication lists, precise time intervals between drug initiation and first skin symptom, de-challenge outcomes, and re-challenge information, are not systematically tabulated or made available in the supplementary materials. Without this granular information, we cannot independently assess confounding by polypharmacy, which is particularly problematic in an elderly population where multiple high-risk drugs (DPP-4 inhibitors, aldosterone antagonists, diuretics) are commonly co-prescribed. Furthermore, the authors do not report what proportion of patients received immunosuppressive therapy (e.g., systemic corticosteroids, doxycycline, rituximab), a key measure of clinical severity and a realistic outcome for future risk-benefit assessments.

The original study lacks transparency regarding the FAERS data search strategy. The authors do not provide specific details on: (i) the exact search terms or query syntax used to identify quinolone-associated reports; (ii) whether the search was restricted to “primary suspect” (PS) drugs only or also included “secondary suspect” and “concomitant” drugs; (iii) the handling of missing or incomplete reports; and (iv) any pre-specified inclusion/exclusion criteria applied beyond the general data cleaning steps described. Without a clearly reported search strategy, the reproducibility of the findings cannot be assured. To facilitate translation of these methodological concerns into actionable research design, **Table 1** summarizes key limitations, their implications, and corresponding future analyses.

**Table 1:** Summary of limitations, implications, and recommended future analyses for quinolone-associated pemphigoid studies.

Limitation in original study	Implication for signal interpretation	Recommended future analysis
No exclusion of concomitant high-risk drugs (DPP-4 inhibitors, aldosterone antagonists)	ROR may be inflated by co-administered drugs	Sensitivity analysis excluding cases with high-risk concomitant medications
Cannot distinguish drug-induced from infection-induced pemphigoid	Signal may reflect underlying infection, not drug	Negative control antibiotics; self-controlled case series
Only nine case reports from literature	Cannot serve as statistical validation	Replication in independent database (EudraVigilance, VigiBase)
No sensitivity analysis for hospitalization rate	Hospitalization may reflect illness burden, not drug-attributable risk	Stratify by quinolone type; compare with/without documented infection
Data-driven quinolone selection (not pre-specified)	Increased risk of type I error (false-positive)	Pre-specify quinolones a priori; adjust for multiple testing
No ICSR-level demographics or concomitant medication lists	Cannot assess confounding by polypharmacy	Provide ICSR-level data as supplementary table (age, sex, concomitant drugs, de-challenge)
No search strategy transparency (terms, PS vs. concomitant, missing data handling)	Findings not reproducible	Report search strategy following READUS-PV guidelines

## 5 The systematically missing role of the clinical pharmacist

The original study lists pharmacist reports as a category (10.9%) but provides no analysis or interpretation. In reality, clinical pharmacists are underutilized sentinels in spontaneous reporting systems. In many hospitals, pharmacists perform daily medication reconciliation, identify potential ADRs, and unlike consumers or even busy physicians, can provide structured, high-quality reports that include drug indication, duration, concomitant medications, and de-challenge/re-challenge information (6–9). The finding that elderly females are at highest risk is actionable for pharmacists. We propose the following concrete, clinically actionable three-step workflow for pharmacists when dispensing a quinolone to a high-risk patient (elderly, female, or already on DPP-4 inhibitors/aldosterone antagonists): Step 1 - Identify: At medication reconciliation or dispensing, flag the quinolone prescription if the patient meets any of the following: age ≥65 years, female sex, concomitant DPP-4 inhibitor or aldosterone antagonist, or prior autoimmune history. Document these risk factors in the patient’s pharmacy record. Step 2 - Assess: Contact the prescriber to discuss two questions: (a) Is a non-quinolone alternative (e.g., beta-lactam, macrolide, nitrofurantoin for uncomplicated UTI) clinically appropriate? (b) If a quinolone is necessary, confirm that the prescriber has documented a baseline skin examination and a monitoring plan. Step 3 - Intervene: Provide structured patient counseling and schedule a pharmacist-led follow-up call at day 7 to inquire about any skin changes.

In addition, the study identifies a short latency period (most cases within 30 days). This is pharmacist-prime territory. Standardized pharmacist counseling text: “You are starting [quinolone name]. In rare cases, this medication can cause a skin reaction called pemphigoid, blisters or skin peeling that may appear within the first month. Please check your skin daily, especially your arms, legs, and trunk. If you see any new blisters, open sores, or severe itching that feels different from a normal rash, stop the medication immediately and go to the nearest emergency department or call our pharmacy at [phone number]. Do not wait for your next dose. This is important even if you have taken this antibiotic before without problems.” The pharmacist should record in the dispensing system that counseling was provided, the date, and the patient’s acknowledgment (signature or electronic confirmation). This type of structured medication counseling is rarely captured in FAERS but represents a real-world risk mitigation strategy. Concrete EHR integration for pharmacists: (1) Automated alert trigger: When a pharmacist enters a quinolone prescription for a patient aged ≥ 65 years or a patient with an active DPP-4 inhibitor or aldosterone antagonist in their medication list, the pharmacy system displays a pop-up alert: “HIGH RISK FOR PEMPHIGOID - Review skin monitoring protocol and document counseling.” (2) Documentation template (structured fields): Pharmacists complete a mandatory form before dispensing, including baseline skin status, counseling date and time, patient acknowledgment, prescriber contacted, and alternative antibiotic discussed. (3) Link to dermatology: If a patient later receives a diagnosis code for bullous pemphigoid (ICD-10 L12.0) or other blistering disorder, the EHR automatically notifies the pharmacy system to flag the patient as quinolone-allergic and adds the reaction to the permanent allergy list. The pharmacist then performs medication reconciliation to remove any standing quinolone orders. By integrating pharmacists into the pharmacovigilance workflow, the field can move from passive, biased spontaneous reports to active, quantified risk assessment.

## 6 A roadmap from signal to causation

Based on the limitations identified above, we propose the following forward-looking recommendations for future research on quinolone-associated pemphigoid. To be clear, the nine case reports in the original study are not a limitation per se; they provide useful supportive qualitative evidence that is broadly consistent with the FAERS findings. However, they cannot, and the original authors do not claim they can, serve as independent statistical validation. The recommendations below are therefore aimed at future studies seeking to move beyond qualitative consistency toward quantitative causal inference. First, findings from FAERS should be replicated in independent spontaneous reporting systems such as EudraVigilance, VigiBase, and regional Chinese ADR databases to assess consistency across different reporting populations and biases. A signal that appears only in FAERS and cannot be replicated elsewhere is likely driven by database-specific biases rather than a true pharmacological effect. Second, negative control designs should be employed, comparing quinolones with other antibiotics (e.g., amoxicillin, azithromycin) for the same event, and using a negative control outcome (e.g., appendicitis) to detect residual confounding that cannot be eliminated by standard adjustment. Third, the self-controlled case series design is particularly suited to short-latency immune-mediated events like pemphigoid, as it automatically controls for time-invariant confounders including genetics, sex, and chronic comorbidities without requiring an external comparator group. Fourth, to translate these epidemiological insights into clinical practice, a pharmacist-led risk scoring tool should be developed and validated at the point of care, incorporating key predictors such as quinolone type, age, sex, concurrent DPP-4 inhibitor use, and prior autoimmune history to guide personalized prescribing decisions. Fifth, electronic health record-based prospective cohorts are needed to link detailed quinolone exposure data (dose, duration, indication) to dermatology and pathology records, with active follow-up for at least 90 days to capture delayed-onset cases. Sixth, any future pharmacovigilance study on quinolone-associated pemphigoid must include sensitivity analyses: (i) exclude cases with concomitant high-risk drugs (DPP-4 inhibitors, aldosterone antagonists, immune checkpoint inhibitors); (ii) stratify hospitalization rates by quinolone type; and (iii) compare hospitalization rates between patients with documented infection and those without. Such analyses would help distinguish drug-attributable risk from illness-related confounders. Future studies should adhere to the READUS-PV guideline, which explicitly recommends transparent reporting of ICSR characteristics.

Finally, and crucially, further studies must close the loop from detection to management: beyond reporting hospitalization rates, they should systematically capture time to diagnosis after first skin symptom, specific treatments received (topical or systemic steroids, doxycycline, immunosuppressants), time to resolution after quinolone withdrawal, and whether the quinolone was permanently added to the patient’s allergy or adverse reaction record. Only through such a comprehensive, causality-oriented, and clinically integrated approach can the field move beyond statistical association toward meaningful patient safety improvement. This last point, closing the clinical loop, is exactly where pharmacists add unique value including documentation in the electronic medical record, medication reconciliation during subsequent admissions, and patient education to avoid inadvertent re-exposure.

In conclusion, Li and colleagues have provided a useful initial signal that quinolone antibiotics are associated with disproportionate reporting of pemphigoid in FAERS. However, translating this signal into clinical action requires addressing profound confounding by co-medication and by indication, moving beyond nine literature cases as the sole source of supportive qualitative evidence, and most critically, integrating the clinical pharmacist into both pharmacovigilance and patient care. A drug safety signal that does not reach the point of prescribing, dispensing, and patient counseling has limited clinical impact. The next generation of studies on quinolone-associated pemphigoid could be pharmacist-informed, actively monitored, and causally robust.
